# A Novel Mammal-Specific Three Partite Enhancer Element Regulates Node and Notochord-Specific *Noto* Expression

**DOI:** 10.1371/journal.pone.0047785

**Published:** 2012-10-22

**Authors:** Leonie Alten, Karin Schuster-Gossler, Michael P. Eichenlaub, Beate Wittbrodt, Joachim Wittbrodt, Achim Gossler

**Affiliations:** 1 Institute for Molecular Biology, Medizinische Hochschule Hannover, Hannover, Germany; 2 Centre for Organismal Studies, University of Heidelberg, Heidelberg, Germany; Instituto Gulbenkian de Ciência, Portugal

## Abstract

The vertebrate organizer and notochord have conserved, essential functions for embryonic development and patterning. The restricted expression of developmental regulators in these tissues is directed by specific cis-regulatory modules (CRMs) whose sequence conservation varies considerably. Some CRMs have been conserved throughout vertebrates and likely represent ancestral regulatory networks, while others have diverged beyond recognition but still function over a wide evolutionary range. Here we identify and characterize a mammalian-specific CRM required for node and notochord specific (NNC) expression of NOTO, a transcription factor essential for node morphogenesis, nodal cilia movement and establishment of laterality in mouse. A 523 bp enhancer region (NOCE) upstream the *Noto* promoter was necessary and sufficient for NNC expression from the endogenous *Noto* locus. Three subregions in NOCE together mediated full activity in vivo. Binding sites for known transcription factors in NOCE were functional in vitro but dispensable for NOCE activity in vivo. A FOXA2 site in combination with a novel motif was necessary for NOCE activity in vivo. Strikingly, syntenic regions in non-mammalian vertebrates showed no recognizable sequence similarities. In contrast to its activity in mouse NOCE did not drive NNC expression in transgenic fish. NOCE represents a novel, mammal-specific CRM required for the highly restricted *Noto* expression in the node and nascent notochord and thus regulates normal node development and function.

## Introduction

The organizer of vertebrate embryos is essential for early embryonic patterning (reviewed in [1]). Additionally, cells of the organizer generate the notochord, a signaling center along the midline of the embryo that patterns surrounding tissues during subsequent development [2]. Development and properties of the organizer are regulated by a network of genes that are expressed in the organizer and its derivatives [3], [4], [5], [6]. “Not” genes constitute a group of homeobox genes that are expressed in the organizer and notochord of vertebrate embryos [7], [8], [9], [10], [11], [12]. The zebrafish Not gene *floating head* (*flh*) and its mouse orthologue *Noto* are pivotal for normal development and function of the organizer and notochord in both species [9], [12]. However, while the function of Not genes in the establishment of asymmetry seems to be retained [13], [14] its role in notochord development has diverged. *Flh* is essential for the formation of the notochord along the entire anterior-posterior body axis [9], [15]. In contrast, *Noto* is required for notochord formation only posterior to the lower trunk region [12]. In addition, *Noto* is essential for morphogenesis of the node, which constitutes the mouse late organizer (reviewed in [5]), for posterior localization of cilia on node cells and nodal ciliogenesis, and thus for the establishment of laterality [13], [16]. Similarly, *flh* function in zebrafish embryos is required for the formation of normal Kupffer’s vesicle [14], a transient structure that functions in the establishment of left-right asymmetry equivalent to the mammalian posterior notochord [17], [18], [19], [20].

*Noto* is first expressed in the mouse embryo during gastrulation in the anterior primitive streak. On embryonic day (E) 7.5 expression is confined to the node, a shallow depression in the outer curvature at the distal tip of the embryo [21], which constitutes the posterior extreme of the forming notochord [22]. During subsequent development *Noto* transcripts are confined to the nascent notochord until axis elongation seizes [12]. Similarly, *flh* is expressed in zebrafish embryos during gastrulation in the notochord precursors, and subsequently in the posterior notochord [9]. However, in contrast to murine *Noto*, which is exclusively transcribed in the node and nascent notochord [11], [12] varying additional sites of Not gene expression were detected in zebrafish, frog and chick embryos [8], [9], [10], [23].

Although the expression patterns of *Noto* and *flh* are similar in mouse and zebrafish embryos during gastrulation the requirement for known transcription factors that function in the node/notochord appears to differ. Mouse embryos lacking T show no *Noto* expression [12], which places T upstream of *Noto*. In contrast, the initial expression of *flh* is independent from the Brachyury homologue *ntl* in zebrafish and just reduced in *ntl* mutants at later stages [9], [23]. Likewise, in mouse embryos *Noto* expression is abolished in embryos lacking FOXA2 [12], a transcription factor essential for node and notochord formation [24]. Whether *flh* expression depends on FOXA2 also in zebrafish has not been reported, but in zebrafish *monorail* mutants that lack *Foxa2* function notochord formation is normal [25], indicating that *flh* is expressed. The promoter region of *Noto* contains numerous FOXA2 binding sites, suggesting that *Noto* is regulated by FOXA2. However, since *Foxa2* mutant mouse embryos lack a node and notochord, the absence of *Noto* expression might merely reflect the absence of *Noto* expressing cells. Thus whether and how FOXA2 contributes to *Noto* and *flh* expression and whether the regulation of these “Not” genes differs between mouse and zebrafish is not clear.

A number of cis-regulatory modules (CRMs) that direct expression of notochord genes have been described in the mouse and zebrafish genomes. They were mainly identified based on reporter gene analyses of sequences highly conserved between vertebrate species in non-coding regions of notochord-expressed genes. Recently, integrating notochord expression data with FOXA2 ChIP-seq data from mouse liver has successfully identified some CRMs in mouse, mediating notochord expression when tested in zebrafish [26], [27], [28], [29]. Many of these CRMs contain a FOXA2 binding site(s) and function by cooperation of FOXA2 with other factors and across species despite the apparent lack of overall sequence conservation [26], [29]. Here, we analyze the proximal enhancer region of murine *Noto*. We delineate a 523 bp module, referred to as NOCE (Node and nascent notOChord Enhancer) about 7 kb upstream of exon1 that is sufficient for node and nascent notochord (NNC)-specific transcription of heterologous genes. NOCE is a functional tissue-specific CRM essential for the expression of endogenous *Noto*. Strikingly, NOCE is conserved in mammalian species but no sequence alignment can be found to the genomes of species outside eutherian mammals. In contrast to other notochord enhancers whose activation in vivo requires functional FOXA2 binding sites [26], [29] a FOXA2 site that activates NOCE in vitro is dispensable for NOCE function in vivo, and NOCE does not function in fish embryos. We also define an orphan binding site (OBS) that is present in a subset of other notochord enhancers and acts in the context of NOCE redundantly with the FOXA2 site. Thus, NOCE constitutes a novel mammal specific cis-regulatory element essential for the regulation of node morphogenesis and function.

## Materials and Methods

### Generation of ES Cells Carrying Single Copy Reporter Gene Insertions at the HPRT Locus

Reporter constructs were introduced by homologous recombination into the genome of E14TG2a ES cells [30]. These cells carry an approximately 35 kb deletion at the HPRT locus and are sensitive to HAT selection. The targeting vector pMP8 [31] restores HPRT expression and HAT resistance in E14TG2a cells upon homologous recombination, thus allowing for direct selection of correctly targeted ES cells. Reporter constructs were introduced into pMP8 upstream of the *Hprt* locus by conventional cloning or InFusion cloning (Clontech; according to manual), linearized with Mfe1 and introduced into E14TG2a ES cells by electroporation. HAT resistant clones were expanded and the integrity of the insertion verified by PCR spanning the 5′ and 3′ homology arm, respectively. For primer sequences see Table S1.

### Generation and Collection of Chimeric Embryos

ES cells carrying different promoter-reporter constructs or the NOCE deletion were injected into wild type morulae and transferred into pseudo-pregnant females. Chimeric embryos (see Table S2 for numbers) were collected at day 7 to 9 after transfer and stained for β-galactosidase activity.

### β-galactosidase Staining and Vibratome Sections

β-galactosidase staining was carried out as described [32]. Embryos were stained for at least 1 hour or over night. For vibratome sectioning embryos were postfixed in 4% PFA over night, briefly washed in PBS and incubated 5 min in 20% gelatine in PBS at 65°C. Subsequently, embryos were transferred into fresh gelatine and oriented on ice until the gelatine hardened. Blocks were fixed over night in 4% PFA and washed in PBS. 80 µm vibratome sections were prepared and mounted in Kaisers glycerol gelatine (Merck).

### Site-directed Mutagenesis

Transcription factor binding sites were mutated using the Quick Change Site-directed Mutagenesis Kit (Stratagene) according to the manufacturer’s instructions. For primer sequences see Table S1.

### Transactivation Assays

Luciferase reporter constructs were generated by introduction of *Noto* upstream sequences into pGL4.27 (Promega). For normalization phRL-TK (Promega) was cotransfected and renilla luciferase activity was simultaneously analyzed. Expression vectors of TCF, LEF and β-catenin were in pCS2+ and a kind gift of Bernhard Herrmann, the Tead and YAP constructs were in pcDNA3.1 and a kind gift of Hiroshi Sasaki. Constructs were introduced into HeLa cells by transfection with Perfectin (GTS, # T303015) according to the manufacturer’s instructions. Luciferase activity was measured with the Dual luciferase reporter assay system (Promega, #E1980) in a Glomax96 luminescence reader.

### Expression Analysis in Fish Embryos

The NOCE element was cloned into the HindIII restriction sites of a a pBlueScript-based transgenesis vector containing two recognition sites for the meganuclease ISce-I [33] flanking a multiple cloning site followed by the hsp70 core promoter [34] GFP and an SV40 polyadenylation signal. In the absence of a functional enhancer, the reporter construct results in GFP expression in the lens, which serves as internal control for successful injections. Injections were done as described previously [35]. DNA was purified using the Maxiprep Kit (Qiagen) and injected at a concentration of 10 ng/ml. A Olympus fluorescent microscope (Olympus MVX 10) was used to examine GFP expression in live embryos. Injected embryos were analyzed at different stages to determine the spatio-temporal pattern of GFP expression. As the hsp70 core promoter is activated by temperature changes, the embryos were kept and examined at constant room temperature. Developmental stages were determined by morphological features as described [36].

### Generation of a NOCE Targeting Construct

For targeting of NOCE in the endogenous *Noto* locus a loxM-pGKprom-EM7-Neo-pGHpA-loxM cassette based on PL452 (kind gift of Nancy Jenkins and Neal Copeland) was generated and introduced by BAC recombineering [37] into BAC108j02 in a way that NOCE was deleted in this BAC. For the retrieval vector a diphtheria toxin A cassette was cloned next to 5′ and 3′ miniarms, that were generated by PCR before. Find sequences in Table S1. The construct was completed by recombineering the respective BAC region with the neomycin/kanamycin selection cassette into the retrieval vector. The construct was linearized with Xmn1 for electroporation.

### Generation of ES Cells Carrying a Deletion of NOCE

ES cells heteroallelic for GFP and RFP tagged *Noto* were generated from matings of *Noto^GFP^* mice [13] with mice carrying a *Foxj1* IRES tdtomato transgene in the *Noto* locus [16]. The NOCE targeting construct was introduced into these ES cells (referred to as *Noto^GFP/RFP^*) by electroporation. Homologous recombination events were detected by PCR spanning the 3′ homology arm (Table S1) and validated by southern blot analysis on both sites with probes 5′ and 3′ to the targeting construct. The targeted allele was determined by Southern blot analysis with the 3′ probe and Xho1 digest.

### Analysis and Quantification of Fluorescence Intensities

Fluorescence of GFP and RFP was analyzed with a Leica DMI6000B microscope. Settings for documentation were established using transgenic heteroallelic *Noto^GFP^* ^/*RFP*^ embryos. Pictures of the node region were taken such that the brightness of the green and red channels were roughly equal (Figure S1). All pictures were taken with the same settings for the respective allele. Fluorescence intensities in the node region were quantified with the Leica LAS AF 2.3 quantification tool. The average background in the red channel in *Noto^GFP^* ^/+^ embryos was subtracted from all other average RFP intensities and the average background in the green channel in *Noto^RFP^* ^/+^ embryos was subtracted from all other average GFP intensities. The ratio of both alleles was calculated using the allele containing NOCE as reference allele (see Table S3). As the fluorescence ratio in transgenic *Noto^GFP/RFP^* embryos reflects equal transcription from both alleles ratios were normalized for this factor.

## Results

### Identification of an Enhancer Region in the *Noto* Locus

**Figure 1:**
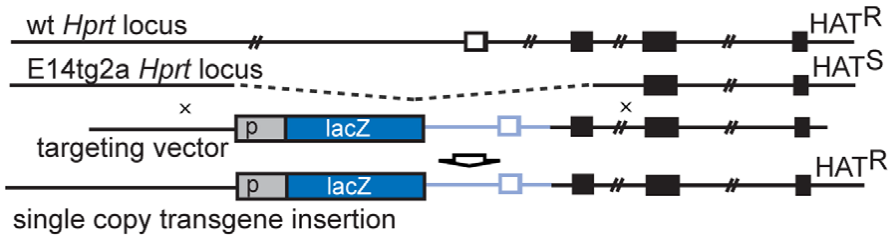
HPRT targeting strategy. Schematic view of wild type and E14tg2a *Hprt* locus, targeting construct and targeted locus. The targeting construct introduces the reporter construct and the promoter and first exon of human *HPRT* to restore *Hprt* function. Wild type and targeted allele are HAT resistant (HAT^R^) whereas the E14tg2a deletion results in HAT sensitivity (HAT^S^).

To identify cis-regulatory elements driving *Noto* expression we first assessed conservation of non-coding sequences from 20 kb upstream of the putative transcriptional start site of *Noto* to the beginning of the next downstream gene *Smyd5* using Multiz alignments [38] and the UCSC Genome Browser database [39] of sequenced vertebrate genomes. The *Noto* upstream region showed conserved regions in the genomes of eutherian mammals, but no conservations were found in the genomes of masurpials and lower vertebrates. In the whole locus investigated, only the second and third exon of *Noto* show some conservation between mammals, birds, amphibians and fish (Figure S2 A). To delineate the region(s) directing the highly restricted NNC expression of *Noto* we analyzed the expression of reporter transgenes in chimeric embryos obtained with ES cells carrying reporter constructs in their genome. To minimize effects of copy number and integration sites on reporter gene expression we introduced by homologous recombination single-copy reporter constructs in the same orientation upstream of the *Hprt* gene (Figure 1; see Mat and Methods for details). This chromosomal region has been shown to allow for faithful expression from various tissue-specific promoters ([40] and e.g. [41], [42], [43]), and chimeric embryos allow for highly reproducible, reliable detection of reporter gene expression with a high efficiency [44], [45]. This strategy ensures the integrity of the construct, allowing for a reliable and efficient comparison in the same genomic context of different promoter-reporter constructs even with only minor size differences.

**Figure 2:**
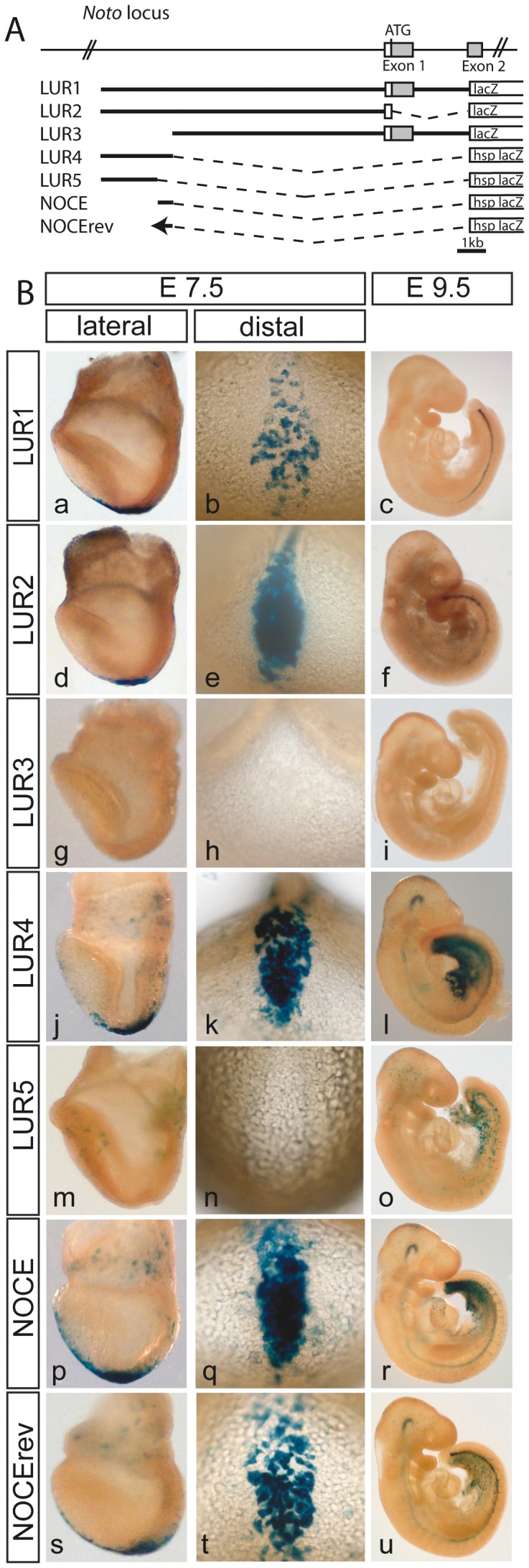
NOCE is necessary and sufficient for reporter gene activity in the NNC. (A) Schematic view of *Noto* upstream regions of various tested transgenes. (B) Corresponding chimeric embryos stained for β-galactosidase activity at E7.5 or E9.5. Transgenes are indicated on the left site.

We started our analysis with a reporter construct, referred to as LUR1 (Long Upstream Region 1) that contained approximately 10 kb upstream the putative start site of *Noto* transcription, exon 1 and intron1, and lacZ (encoding β-galactosidase) fused in frame into exon 2 (Figure 2 A and Figure S2 B). This genomic region of *Noto* was selected based on the presence of 23 putative FOXA2 bindings sites (Figure S2 C) that were determined based on the positional weight matrix of FOXA2 from the JASPAR database [46]. The construct LUR1, as well as a construct LUR2, from which the coding part of exon1 and intron1 were deleted (Figure 2 A), resulted in robust β-galactosidase staining in the NNC (Figure 2 B a–f). β-galactosidase staining in E9.5 chimeras extended into more anterior regions of the notochord than *Noto* transcripts, which most likely reflects the perdurance of the β-galactosidase protein. Deletion of 2.5 kb from the 5′ end of LUR1 (LUR3) completely abolished lacZ expression in chimeric embryos (Figure 2 B g–i), suggesting that sequences essential for driving *Noto* transcription reside in this region. To test whether the 2.5 kb region is also sufficient for NNC expression this region was cloned in front of the lacZ gene fused to the hsp68 minimal promoter [45]. This 2.5 kb construct (LUR4) resulted in strong lacZ expression in the NNC (Figure 2 B j–l) indicating that regulatory elements in this DNA fragment are sufficient for NNC expression. To further delineate the regulatory sequences directing NNC expression we first deleted an approximately 0.5 kb region from the 3′ end of the 2.5 kb fragment and the resulting 2 kb fragment-reporter fusion (LUR5) did not show any lacZ expression in the NNC (Figure 2 B m–o). However, the deleted 0.5 kb fragment alone (from hereon referred to as NOCE (Node and nascent notOChord Enhancer) in front of the hsp68-lacZ reporter led to robust NNC expression in its original genomic or reverse orientation (Figure 2 B p–u) virtually indistinguishable from LUR4 (the 2.5 kb fragment). Thus, NOCE appears to contain sequences that are sufficient to direct NNC expression of the lacZ reporter in the context of a heterologous promoter, irrespective to its orientation towards the promoter (which is assumed to be a general feature of distal cis-regulatory elements).

With the reporter constructs containing the hsp68 promoter additional or increased ectopic expression was obtained on E7.5 in extraembryonic tissues and the streak region, and on E9.5 in the ventral diencephalon, the posterior hindgut, umbilical cord region, intersomitic vessels and scattered cells throughout the embryo. Ectopic expression can be attributed to the hsp promoter, because this ectopic expression was not observed in a construct that contained NOCE in front of approximately 400 bp upstream of *Noto* exon1 as a minimal promoter (Figure S3). Since the ectopic expression in extraembryonic tissues served as a useful indicator for the contribution of ES cells to embryos without specific NNC staining (as observed for example in LUR5), additional constructs were made in the context of the hsp68 minimal promoter (see below).

### NOCE Regulates Endogenous *Noto* Expression

**Figure 3:**
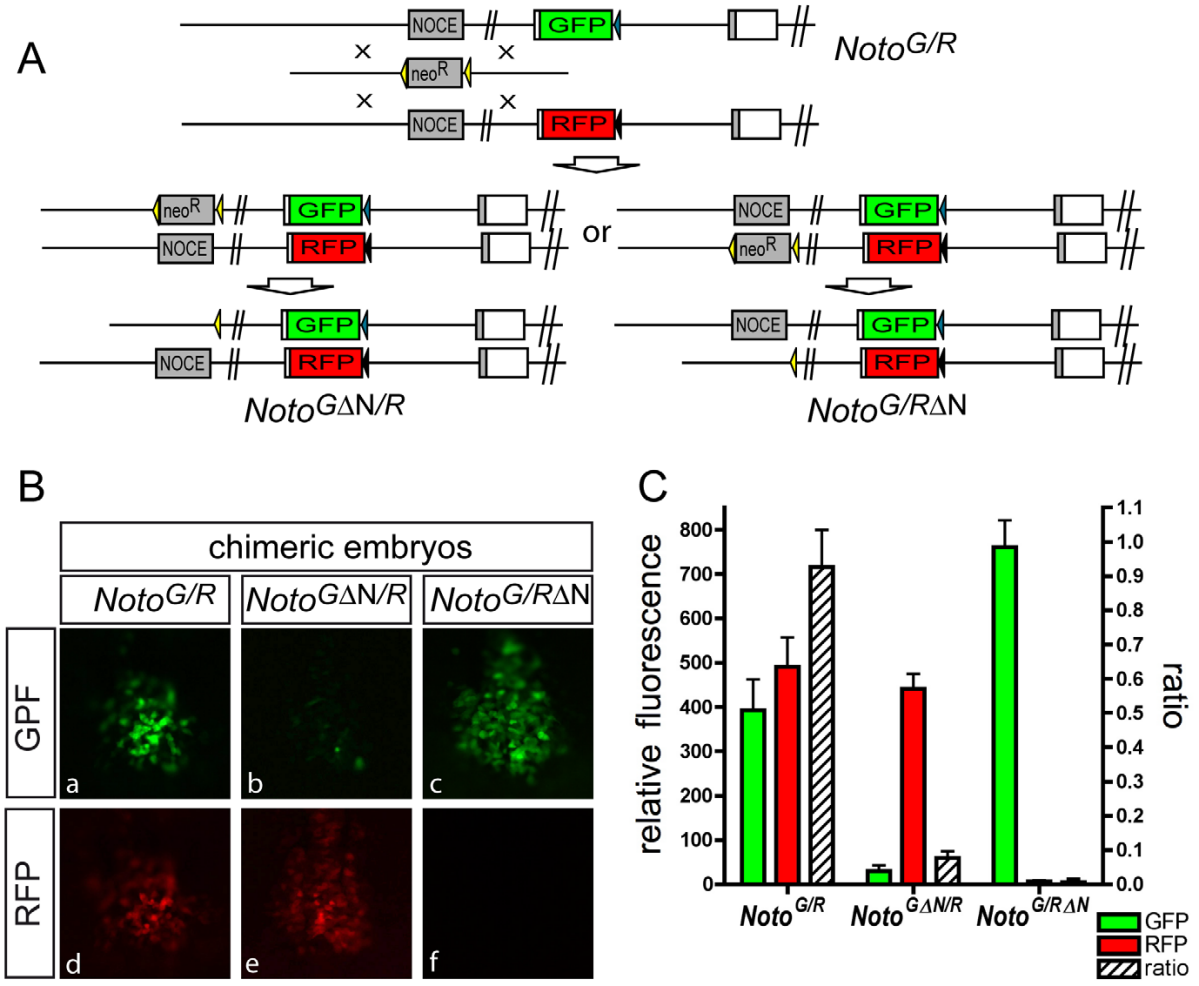
NOCE regulates endogenous allele-specific *Noto* expression. (A) Schematic view of the targeting strategy to delete NOCE in ES cells carrying alleles with green or red fluorescent proteins (GFP and RFP) in the *Noto* locus, respectively. (B) fluorescence of GFP and RFP in chimeric embryos with the parental heteroallelic cell line (B a, d), and cells in which NOCE was deleted from either the GFP (B b, e) or RFP allele (B c, f). (C) Fluorescence intensities of GFP and RFP and ratio of GFP/RFP of control chimeras, and of targeted/non-targeted (reference) alleles for chimeric embryos with one deleted NOCE allele (see also material and methods and Figure S1 and Table S3).

To test whether NOCE directs NNC expression of the endogenous locus we deleted one copy of NOCE by homologous recombination in ES cells that had one allele of *Noto* tagged with GFP and the second allele tagged with tdtomato (*Noto^GFP/RFP^*; Figure 3; see Materials and Methods for details). NOCE was deleted from either allele, to generate *Noto^GFPΔNOCE/RFP^* and *Noto^GFP/RFPΔNOCE^* ES cells, respectively. Chimeric embryos generated with these cell lines were analyzed for GFP and RFP expression at E8 using the fluorescence of the non-targeted allele as a control. Green and red fluorescence intensities were calibrated using heteroallelic *Noto^GFP/RFP^* embryos and their ratio set to one in these embryos (see Materials and Methods, Figure S1 and Table S3 for details). Chimeras obtained with *Noto^GFP/RFP^* ES cells had nodes that expressed GFP and tdtomato (Figure 3 B) at a fluorescence intensity ratio of 0.95. (Figure 3 C, and Table S3). In chimeras obtained with *Noto^GFPΔNOCE/RFP^* cells the GFP signal was severely reduced (Figure 3 B b, e) resulting in a fluorescence intensity ratio of 0.08 (Figure 3 C). In chimeras obtained with *Noto^GFP/RFPΔNOCE^* cells the RFP signal was virtually abolished (Figure 3 B c, f) resulting in a fluorescence intensity ratio of 0.007 (Figure 3 C). The apparent difference in the remaining expression levels can be attributed to differences in the fluorescent proteins in the respective tagged allele (e.g. photostability [47], or translational efficiency (endogenous ATG versus IRES sequence)). The difference in GFP levels between *Noto^GFP/RFP^* and *Noto^GFP/RFPΔNOCE^* likely reflect different degree of chimerism obtained with the different cells lines. Collectively these findings indicate that NOCE is not only sufficient to drive NNC expression, but is also an essential cis-regulatory element required for expression of endogenous *Noto.* This makes NOCE a “bona fide” functional enhancer with biological relevance in a developmental process involved in embryonic patterning.

### NOCE Responds to FOXA2, TEAD and TCF/LEF in vitro but Binding Sites of these Factors are Dispensable for NNC Expression in vivo

**Figure 4:**
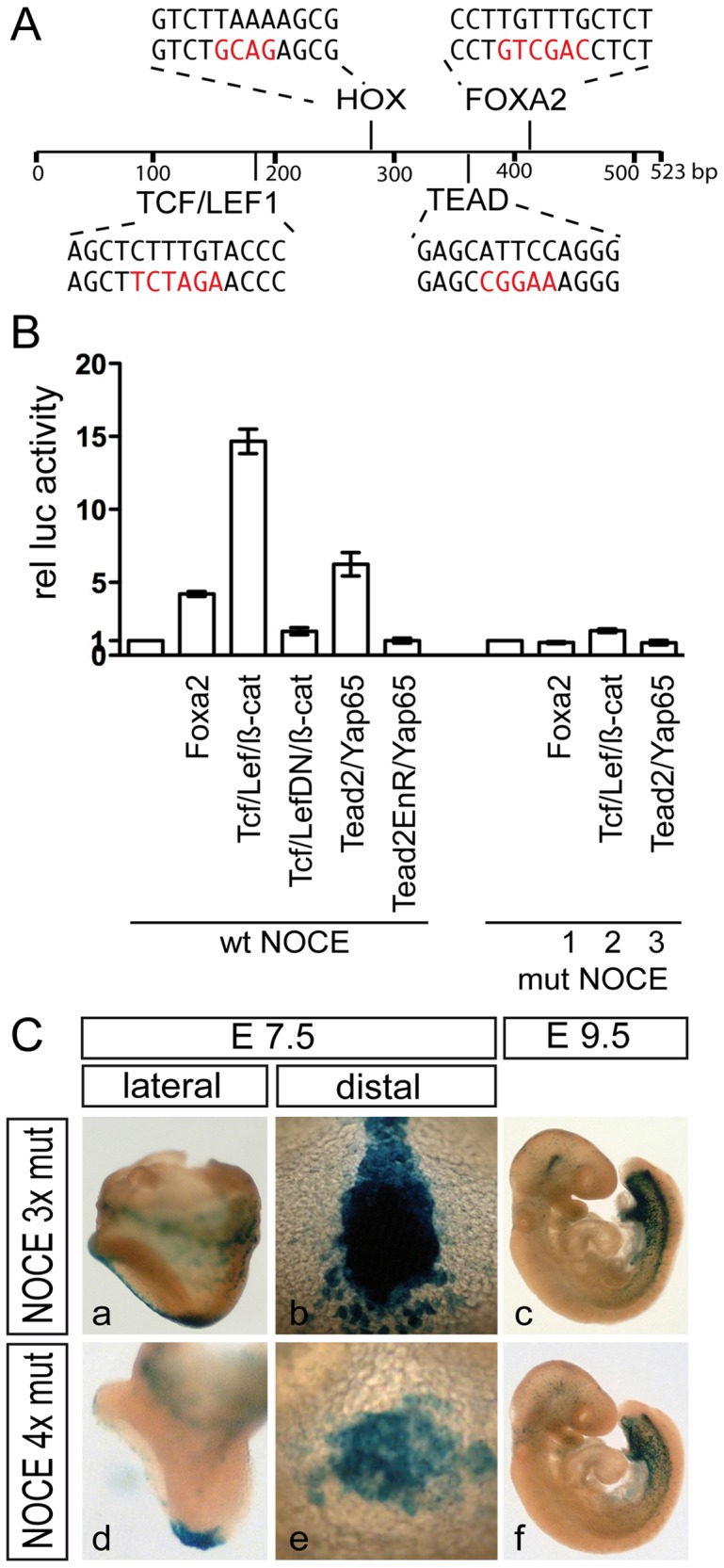
NOCE responds to FOXA2, TEAD and TCF/LEF in vitro, but binding sites are dispensable in vivo. (A) Schematic view of location and sequences of transcription factor binding sites in NOCE. Red characters indicate exchanged nucleotides of mutated sites. (B) Transactivation of wild type and mutated NOCE constructs. Arabical numbers indicate different mutated binding sites: (1) FOXA2, (2) TCF/LEF, (3) TEAD. Cotransfected constructs of transcription factors are indicated below. (C) β-galactosidase staining of chimeric embryos carrying promoter-reporter transgenes with mutated FOXA2, TEAD and TCF/LEF binding sites (NOCE 3x mut) and an additional mutation in the HOX binding site (in NOCE 4x mut) in NOCE.

Multiz alignments [38] detected orthologous sequences to NOCE in the genome of mammalian species (Figure S4). Search for transcription factor binding sites (TFBS) in NOCE (http://www.genomatix.de/) identified one high scoring binding site for FOXA2, TEAD and TCF/LEF1 proteins, respectively (Figure 4 A). As FOXA2 is pivotal for node and notochord development [24], TEAD proteins activate *Foxa2* transcription in the node [27], and a LEF/TCF/β-catenin complex is active in node cells [48], [49] these proteins represented appealing factors that regulate NOCE activity and *Noto* expression in the node. In addition, NOCE contains a HOX binding site raising the possibility that NOTO itself impinges on its regulation. To test whether NOCE responds to these factors in vitro we generated luciferase reporter constructs with NOCE and analyzed the activation of the reporter by the above mentioned factors in HeLa cells. Expression of LEF/TCF/β-catenin, TEAD factors/YAP65, or FOXA2 activated the NOCE-luciferase reporter (Figure 4 B) whereas NOTO had no effect (data not shown). The specificity of reporter gene activation by the above factors was confirmed by expression of dominant negative proteins in the case of LEF and TEAD2, or mutations in the transcription factor binding sites (indicated in red in Figure 4 A), both of which effectively abolished induction (Figure 4 B). To test whether these binding sites are critical for NNC expression in vivo in the embryo we generated ES cells carrying hsp68-lacZ reporters with mutated binding sites. First, we tested the requirement for the TEAD, TCF/LEF1 and HOX binding sites by mutating these sites in the context of the 2.5 kb element including NOCE sufficient for NNC expression (LUR4 see Figure 2 B j–l). Neither a mutated TCF/LEF1 or HOX binding site affected β-galactosidase staining in the NNC (data not shown). Also LUR4 with a mutated TEAD site or all three sites mutated simultaneously resulted in strong β-galactosidase staining in the NNC, but β-galactosidase staining did not extend as much anteriorly in the notochord as we observed with wild type LUR4 (data not shown), which most likely reflects reduced expression levels. Virtually identical staining to the wt construct was observed with a NOCE reporter construct carrying mutations in these three binding sites (Figure 4 C a–c). A NOCE reporter in which the FOXA2 site was mutated in addition still led to readily detectable β-galactosidase staining in the NNC (Figure 4 C d–f). However, β-galactosidase staining did not extend as much anteriorly in the head process and notochord as we observed with wild type NOCE (compare Figure 2 B r with 4 C f and Figure S5 F), suggesting reduced expression levels. In summary, binding sites for LEF/TCF/β-catenin, and HOX appear to be dispensable for NOCE function in vivo, and the TEAD factors/YAP65, or FOXA2 site contribute to full expression levels but are not essential for NNC-specific transcription in the context of the reporter gene.

### Three Enhancer Subregions Contribute to NOCE Activity

**Figure 5:**
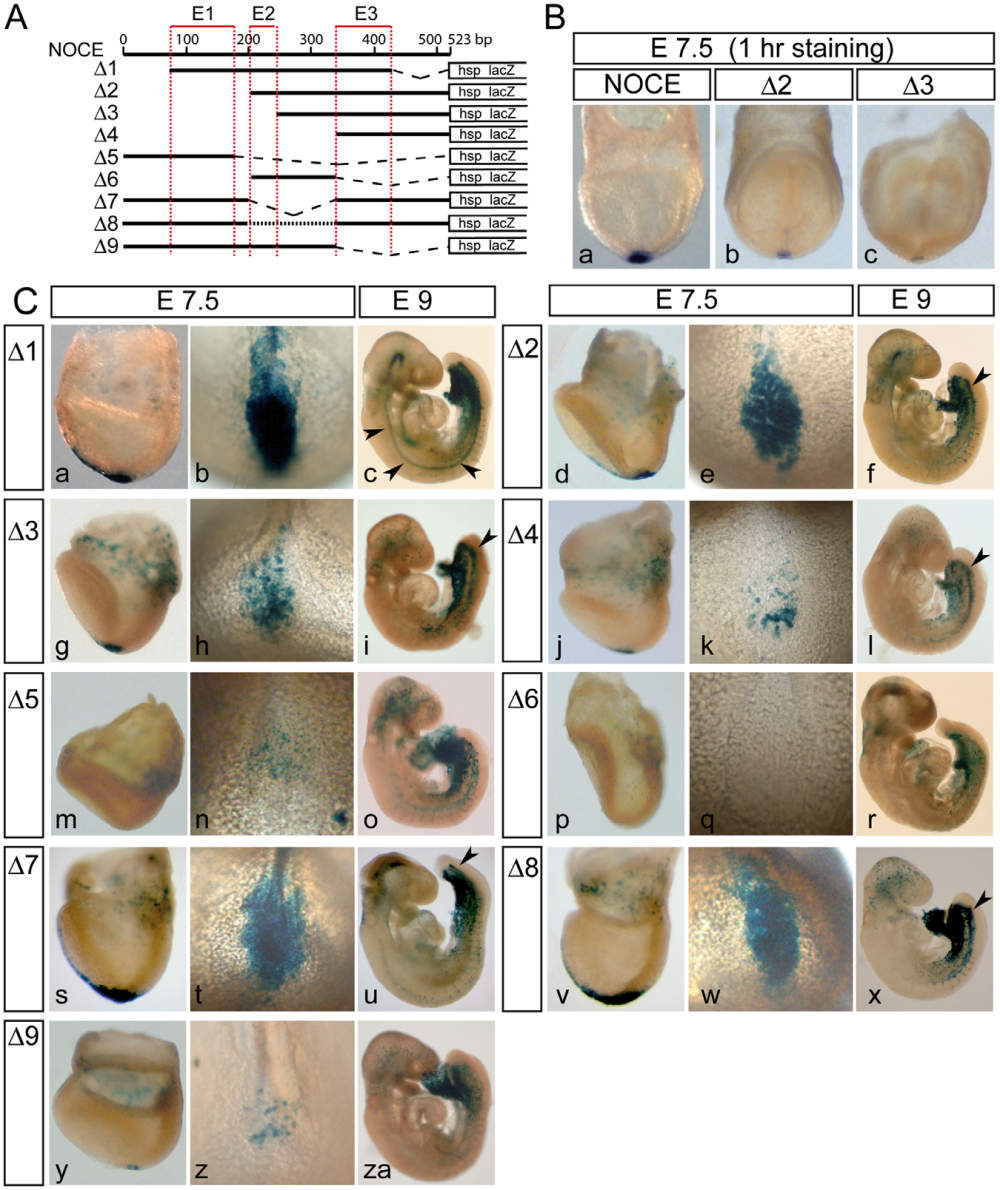
NOCE is a three partite enhancer element. (A) Schematic view of transgenes containing parts of NOCE. (B) 1 hr β-galactosidase staining of different promoter-reporter transgenes in chimeric embryos. (C) Over night β-galactosidase staining of chimeric embryos carrying the transgenes depicted under (A). The stage is indicated at the top and the transgene at the left site. Arrow heads point at stained notochords.

To further define the regions in NOCE that are important for NNC expression we analyzed a set of constructs in which parts of NOCE were deleted (Figure 5 A). Deletion of approximately 100 bp from either end of NOCE (Δ1) resulted in robust NNC expression indistinguishable from full length NOCE (Figure 5 C a–c, and Figure S5 A), reducing the NOCE core sequence to 360 bp. A construct carrying the approximate proximal (3′) two-thirds (Δ2) or the proximal half (Δ3) of NOCE also resulted in NNC staining (Figure 5 C d–i, and Figure S5 B, C). However, *lacZ* expression levels were significantly reduced, which became evident by comparison of chimeric NOCE-lacZ and Δ2 or Δ3-lacZ embryos after brief β-galactosidase staining (Figure 5 B). Higher β-galactosidase activity with the Δ2 construct compared to the Δ3 construct suggested that the small region designated enhancer region E2 (E2) (Figure 5 A) contains sequences that enhance expression levels. Reduced expression levels of constructs Δ2 and Δ3 were also reflected by the restriction of β-galactosidase staining to the nascent notochord compared to the fully active Δ1 construct (compare Figure S5 A-A3 with B-B3 and C-C3). Also the proximal third of NOCE (Δ4) resulted in clear NNC staining after over night color development similar to Δ3 (Figure 5 C j–l and Figure S5 D), indicating that the approximately 100 bp region designated enhancer region E3 (E3) (Figure 5 A) contains sequences sufficient for weak NNC expression. Deletion of two-thirds of the proximal region (Δ5) resulted in very weak but reproducible node staining (Figure 5 C m–o) without clear staining in the nascent notochord on embryonic day 9. This indicated that also this region designated E1 contains regulatory elements that result in weak NNC expression. The central portion of NOCE on its own containing E2 (Δ6) did not direct NNC-specific staining (Figure 5 C p–r, and Figure S5 E). Constructs carrying a deletion of the central portion (Δ7) or in which the central portion was replaced with unrelated non-coding sequences of the same length (Δ8) showed robust NNC expression (Figure 5 C s–x), although β-galactosidase staining in the notochord was restricted to the nascent portion. This suggested some reduction of expression, consistent with the notion that E2 contains sequences enhancing the activity of NOCE. Finally, a construct encompassing the distal two-thirds of NOCE (Δ9) showed specific but weak node expression (Figure 5 C y–za). In conclusion, these results indicated that the distal and proximal portions of NOCE referred to as E1 and E3 (Figure 5 A) contain regulatory sequences that individually can drive weak node and NNC expression, respectively, both together drive robust NNC expression, and E2 further enhances expression levels to full NOCE activity, but is inactive on its own, at least in the context of the heterologous reporter assay.

### An Orphan Binding Site in E1 Acts Redundantly with the FOXA2 Site in E3

**Figure 6:**
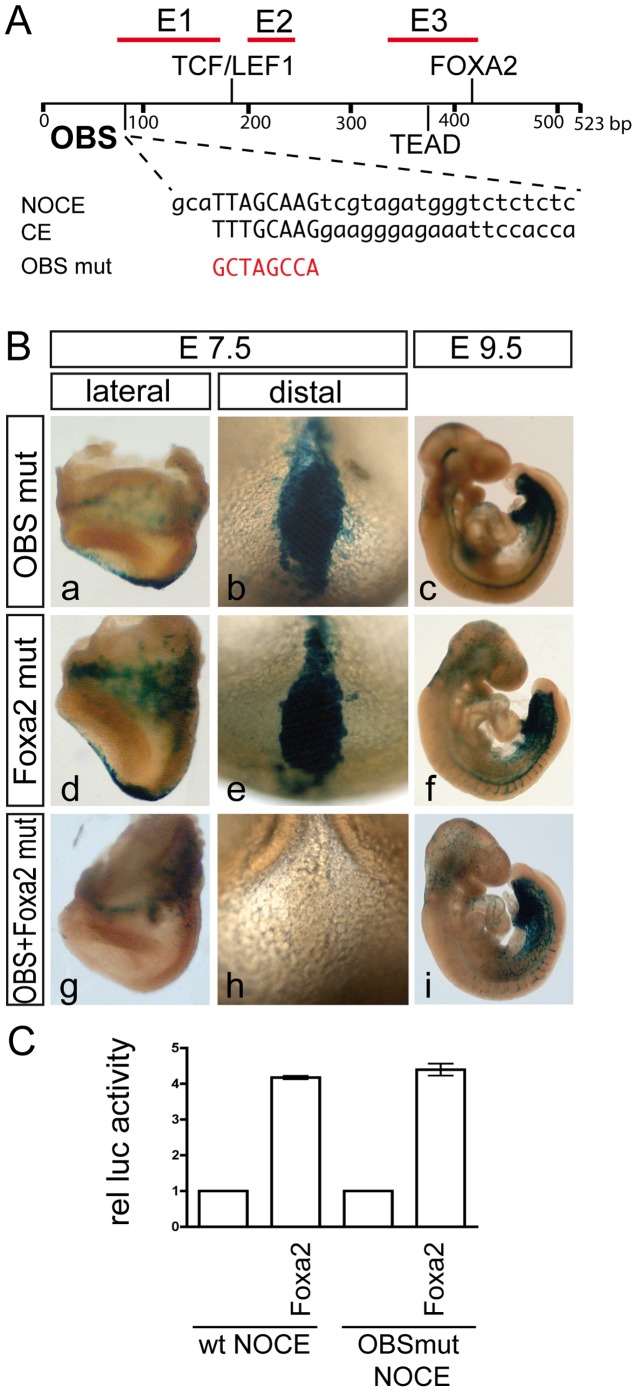
An orphan binding site together with a FOXA2 binding site are essential for NOCE function. (A) Schematic view of location and sequences of transcription factor binding sites in NOCE and the alignment of OBS to CE [27]. Red characters indicate exchanged nucleotides in mutated sites. (B) β-galactosidase stained chimeric embryos carrying a promoter-reporter transgene of NOCE with mutated OBS and/or FOXA2 binding sites. Transgenes are indicated at the left site and the stage at the top.

The enhancer region E3 of NOCE contains the FOXA2 and TEAD binding sites that are present in NOCE, whereas in silico analyses of E1 and E2 did not reveal binding sites for known transcription factors associated with transcriptional regulation in the NNC. However, comparison of the E1 sequence with the core sequence (CE) from the known mNE enhancer of the *Foxa2* gene driving node expression [27], [50] showed one 8 bp region (from hereon referred to as OBS (Orphan Binding Site)), which resembled with one mismatch the 5′ part of the core enhancer (CE) element of *Foxa2* mNE enhancer (Figure 6 A and Figure S6) that is essential for expression in the node [27]. This raised the possibility that OBS is a site in E1 that mediates E1 function. The single mutation of OBS in NOCE had no obvious effect on NNC expression (Figure 6 B a–c, and Figure S5 G) in contrast to a mutation of the FOXA2 site, which led to slightly reduced expression (Figure 6 B d–f, and Figure S5 F). However, when both OBS and the FOXA2 site were mutated in a NOCE reporter, NNC expression was completely abolished (Figure 6 B g–i), indicating that both sites together are essential and function redundantly to establish NOCE enhancer activity. To test whether OBS despite its sequence divergence from a bona fide FOXA2 site might be a target of FOXA2 we generated a luciferase reporter construct with NOCE carrying a mutated OBS and tested its response to FOXA2. This construct responded to FOXA2 indistinguishable from the wild type NOCE reporter (Figure 6 C), indicating that OBS does not contribute to NOCE activity through binding of FOXA2.

### NOCE is Inactive in Fish Embryos

In a recent study to identify FOXA2 regulated genes expressed in the notochord, seven novel enhancers were identified in the mouse genome that directed heterologous gene expression in the notochord of zebrafish embryos. Only two of them are sequence conserved from mouse to fish, while the remaining five are only conserved among mammalian species [29]. Additionally, the known mouse notochord enhancers *Foxa2* mNE [27], [50] and *Sox9* E1 [51] function in fish embryos [26], [29]. Therefore, we tested whether NOCE might similarly be active in medaka fish embryos. We used an HSP70 based fish expression vector that exhibits control GFP activity in the developing lens of successfully injected zygotes [52] and analyzed the enhancer activity of NOCE in developing medaka embryos. Upon injection at the one cell stage, 221 embryos exhibited control activity in the developing lens. However none of these embryos exhibited significant activity outside of the control region, in particular not in the forming or developing notochord (Figure S7). Thus the NOCE domain cannot interact with transcription factors in fish embryos to activate NNC specific reporter expression, suggesting that the NOCE represents an element that is specific for higher vertebrates.

## Discussion

Our analysis using single copy transgenes in a specific chromosomal context has defined a 523 bp cis regulatory region of the *Noto* gene, NOCE, that is conserved between mammalian species, sufficient to direct heterologous gene expression in the NNC, and is critically important for transcription of endogenous *Noto,* indicating that this cis-regulatory element is a functional *Noto* enhancer. The NOCE 360 bp core region contains three enhancer subregions whose cooperation is required for full activity. NOCE activity depends on a FOXA2 binding site and an orphan factor binding site, OBS. In the context of NOCE the presence of OBS or the FOXA2 site is sufficient for enhancer activity. However, when these sites are mutated simultaneously enhancer activity is lost, indicating that FOXA2 and the factor binding to OBS act redundantly in directing NNC-specific *Noto* expression in mammalian embryos.

### NOCE is a Functional Tri-partite Enhancer Regulating Endogenous *Noto* Expression

Deletion of NOCE from one allele in ES cells that had each *Noto* allele labeled by expression of a different fluorescent protein resulted in severe downregulation of the targeted allele. Thus, our deletion analyses showed that *Noto* transcription depends critically on the presence of NOCE, and define NOCE as a pivotal regulatory element directing highly restricted *Noto* expression. Three enhancer subregions with different properties interact and contribute to full NOCE activity. Spatio-temporal specificity is not confined to a single subregion, but the regions rather interact to establish robust expression. Region E1 directed weak expression in the node, which was enhanced by the presence of region E2. Region E3 directed more robust expression in the NNC, which was also enhanced by the presence of E2. Regions E1 and E3 either with the native spacing or juxtaposed resulted in robust NNC expression, which was further enhanced by the presence of E2. Thus, E1 and E2 both appear to independently cooperate with E3, which on its own is the strongest of the three regulatory subregions, and the synergistic action of the three subregions is required for full NOCE activity. Strikingly, the two NOCE subregions which show activity in the NNC direct expression of the full pattern albeit at reduced levels. Synergism between different transcription factor binding sites is generally observed in enhancer regions [53] and has been described for various other notochord enhancers [26], [27], [29], [54]. Since the deletion of E1 from NOCE has a more severe effect than the mutation of OBS (compare Fig 5 C d–f with 6 B a–c), and deletion of E3 affects NNC expression levels much more than mutation of the FOXA2 site (compare Fig 5 C y–za with Fig 6 B d–f ), it is highly likely that additional binding sites in the enhancer subregions E1 and E3 are required for the synergistic activity of the three enhancer subregions.

The presence of a TCF/LEF, TEAD and FOXA2 binding site in NOCE, the known activity of these factors in node cells [27], [29], [49], [55], and the activation of a NOCE-driven luciferase reporter by these factors in cultured cells (Figure 3 B) suggested that these TFBS contribute to the activity of NOCE. Surprisingly, neither mutations in individual binding sites, nor their combined disruption significantly affected NOCE-driven reporter gene expression in vivo, indicating that these factors do not contribute to NOCE activity or the loss of their binding sites can be compensated.

A major role of FOXA2 was suggested by loss of *Noto* expression in embryos lacking FOXA2 [11], [12], as well as the presence of FOXA2 binding sites in the region 10 kb upstream the putative start site of *Noto* transcription and in the first intron, and a pivotal role of FOXA2 binding sites for the function of other notochord enhancers [26], [29]. However, the FOXA2 sites in the upstream region of *Noto* outside of NOCE (constructs LUR3 and LUR5) are not sufficient for lacZ reporter expression in the NNC. Furthermore, the deletion of NOCE from the *Noto* locus eliminated endogenous expression despite the remaining numerous FOXA2 sites, indicating that binding of FOXA2 alone is not sufficient. We do not know whether these sites are occupied by FOXA2 in node cells. However, there are reported ENCODE-ChIP-data from a human liver carcinoma cancer cell line (HepG2) [56] showing FOXA2 binding around 2 kb upstream of the *Noto* gene and to the human NOCE region (chr2∶73,418,686-73,419,171 in hg19 assembly), indicating FOXA2 occupancy of some of these sites. Nonetheless our findings indicate that FOXA2 is not sufficient to activate the *Noto* promoter both in the context of our reporter constructs and the endogenous locus. Thus, the absence of *Noto* expression in *Foxa2* mutants rather reflects the absence of a distinct node than a strict requirement of FOXA2 protein binding to NOCE.

FOXA proteins act as ‘pioneer factors’ whose binding to promoters and enhancers enables chromatin access for other tissue-specific transcription factors [57]. Thus, it is possible that FOXA2 is a permissive factor enabling other factors that bind upstream of LUR3 to stimulate *Noto* expression, which is consistent with the activity of LUR1 and LUR2. However, in contrast to other experimentally validated notochord enhancers in which FOXA2 sites are essential [26], [29] the single FOXA2 site in NOCE, which responded to FOXA2 in cultured cells, was not required for NOCE activity in vivo (Figure 4 and 6), and mutation of OBS did not affect NOCE activation by FOXA2 in vitro (Figure 6 C), indicating that another protein(s) can compensate the loss of FOXA2 binding. A good candidate for this compensatory factor is a protein that can bind to OBS, because when the OBS and FOXA2 sites were mutated simultaneously the enhancer function was completely lost. Thus, NOCE is composed of enhancer elements that act cooperatively, and of others that have equivalent or redundant functions, but are bound by proteins with different sequence specificity.

OBS is highly similar to the 5′ part of the core enhancer (CE) element of the *Foxa2* gene and might bind the same factor(s). The 5′ CE sequence is essential for *Foxa2* expression in the node and notochord [27]. Thus, it is possible that FOXA2 together with a factor that regulates *Foxa2* expression and has similar functions, impinge on NOCE and regulate its activity. While the 3′CE sequence has been shown to be bound by TEAD, the 5′ CE sequence was suggested to bind to an unknown factor termed POT (Partner Of TEAD), which might also bind to OBS and could not be identified in Yeast-one-hybrid screens [27]. Also our attempts to identify and validate a protein that binds to OBS by Yeast-one-hybrid screening were not successful (data not shown). However, we found multiple occurrences of the OBS/CE 8 bp-core motif TTAGCAAG/TTTGCAAG with allowing one mismatch in the experimentally validated mouse notochord enhancers of *Foxa2* [27], [50], *Sox9* [51], and in 4 of the 7 validated notochord enhancers reported by the study of Tamplin [29]. This suggests that the OBS/CE element constitutes a feature of a subtype of notochord enhancers and plays a more general role in the regulation of genes expressed in the node and notochord.

### NOCE Constitutes a Mammalian Species-specific “Not Homeobox” Enhancer

Comparison of NOCE and the *Noto* upstream region with vertebrate genome sequences detected sequences highly similar to NOCE in a similar location upstream of *Noto* exon 1 in eutherian mammals but not in the upstream regions of *Noto* orthologues in avian, amphibian or fish genomes. The expression domains of murine *Noto* and its orthologues in lower vertebrate species (*flh*, *Xnot1/2, Cnot1/2*) differ in that *Noto* expression in mouse embryos is confined to the node/nascent notochord during gastrulation and axis elongation, whereas varying additional sites of expression such as anterior neuroectoderm, epiphysis, limb bud and foregut were detected in zebrafish, *Xenopus* and chick embryos [8], [9], [10], [23]. Thus, the unique presence of NOCE in mammalian genomes correlates with and may be the basis for the more restricted expression pattern in these species.

A number of previously identified notochord-specific mouse enhancers are not sequence conserved to zebrafish but drive reporter gene expression in the notochord of zebrafish embryos [29]. This suggests, that the (transcription-) factors regulating these enhancers remained conserved from mouse to fish. This could indicate either mutation of functionally conserved enhancers beyond sequence recognition or a (rapid) turnover of enhancers utilized in teleosts. Either possibility points to a high flexibility of cis-regulatory elements driving notochord-specific expression. The highly specific NOCE enhancer characterized in this study activates the reporter gene in a mammal-specific manner in a restricted expression domain. The NOCE enhancer is not conserved between mouse and fish and is not activated by fish transcription factors, which indicates that NOCE belongs to a group of NNC enhancers that have evolved with the emergence of mammals, and suggest that the regulation of a subset of genes involved in node/notochord formation and function has significantly diverged during vertebrate evolution.

In summary, our analysis has defined a novel cis-regulatory element, NOCE, that directs the highly specific and restricted expression of the homeobox gene *Noto.* Binding of FOXA2 or of a factor binding to OBS that acts redundantly with FOXA2 is key to transcription of *Noto*. The combination of OBS and FOXA2 sites in other validated notochord enhancers suggests that this is a general feature of some mammalian notochord enhancers. NOCE function appears to be specific for mammalian species in contrast to a number of other notochord enhancers, indicating that the regulation of Not type homeobox genes, despite or because of their crucial importance in key developmental processes shaping the embryo has considerably diverged between mammals and lower vertebrates.

## Supporting Information

Figure S1 **GFP and RFP fluorescence intensities in transgenic embryos.** (A) GFP and RFP fluorescence in the node of *Noto^GFP/RFP^* (a, d), *Noto^RFP/+^* (b, e) and *Noto^GFP/+^* (c, f) transgenic embryos, (B) Fluorescence intensities of GFP and RFP and the ratio of GFP/RFP and RFP/GFP of transgenic embryos of the indicated genotypes (see also material and methods and Table S3).
(JPG)

Figure S2 **Conservation of the mouse** ***Noto*** **locus and construct LUR1.** (A) Mouse *Noto* locus from the UCSC genome browser (Raney, Cline et al. 2011) with 30-way Multiz alignments (Blanchette, Kent et al. 2004) indicating conservation of sequences within vertebrate genomes. Genome coordinates from NCBI37/mm9 assembly (July 2007). (B) Genomic coordinates (mm9) of LUR1 reporter construct and 30-way Multiz alignments. (C) Position weight matrix logo for the transcription factor FOXA2 from the JASPAR database (Sandelin, Alkema et al. 2004) and location of putative FOXA2 binding sites in the LUR1 construct (85% threshold score). Annotation of UTR, exons and intron are adapted from Ensembl gene annotations.
(JPG)

Figure S3 **Comparison of reporter gene expression obtained by NOCE with the hsp and endogenous** ***Noto*** **minimal promoter.** β-galactosidase staining of chimeric embryos carrying a promoter-reporter transgene of NOCE in front of the minimal promoter from hsp68 (A–C) or the endogenous *Noto* (D–F).
(JPG)

Figure S4 **Alignment of mouse NOCE and orthologous sequences.** Genomic coordinates of mouse NOCE (mm9), Multiz alignment of NOCE to orthologous sequences in representative species and annotation of putative binding sites for TCF/LEF1, TEAD, FOXA2 and HOX. No alignment can be found to species outside eutherian mammals, e.g. marsupial mammals, birds, amphibia and fish. The alignment was done by retrieving the 46-way Multiz hg19 alignments for selected species.
(JPG)

Figure S5 **Gelatine sections of chimeric embryos after β-galactosidase staining.** Genotypes are indicated on the left site. Lines indicate the respective section plane.
(JPG)

Figure S6 **Annotated mouse NOCE core sequence.** Genomic coordinates of mouse minimal-NOCE (mm9), Multiz alignment of the mouse sequence to orthologous sequences of representative eutherian mammalian species and annotation of putative binding sites OBS, OBS_core, TCF/LEF1, TEAD and FOXA2. The enhancer regions E1–E3 are indicated as black bars above the alignment. The alignment was done by retrieving the 46-way Multiz hg19 alignments for eutherian mammals.
(JPG)

Figure S7 **NOCE shows no specific enhancer activity in fish.** Representative medaka embryo, transient transgenic with the NOCE::GFP reporter construct. The control expression in the lens (arrow) is attributed to the activity of the *hsp70* promoter fragment that serves as technical control for successful genomic integration of the reporter. Additional green spots correspond to non-specific ectopic expression occurring in transient injected embryos. The observed autofluorescence (yellow patches) corresponds to the natural chromatophores in medaka fish. Live medaka stage 28 embryo is shown in dorsal view; anterior is oriented to the left.
(JPG)

Table S1 **Sequences of primers used for ES cell screening and verification of promoter-reporter transgene insertions into the** ***Hprt*** **locus.**
(PDF)

Table S2 **Number of analyzed chimeric embryos with various promoter-reporter transgenic ES cells.**
(PDF)

Table S3 **Fluorescence intensities and ratios of transgenic and chimeric embryos.**
(PDF)
