# Supplementary figures and images for: A Novel Mammal-Specific Three Partite Enhancer Element Regulates Node and Notochord-Specific Noto Expression
Source: PLoS One. 2012 Oct 22;7(10):e47785. doi: 10.1371/journal.pone.0047785 (PMC3478275; doi:10.1371/journal.pone.0047785)

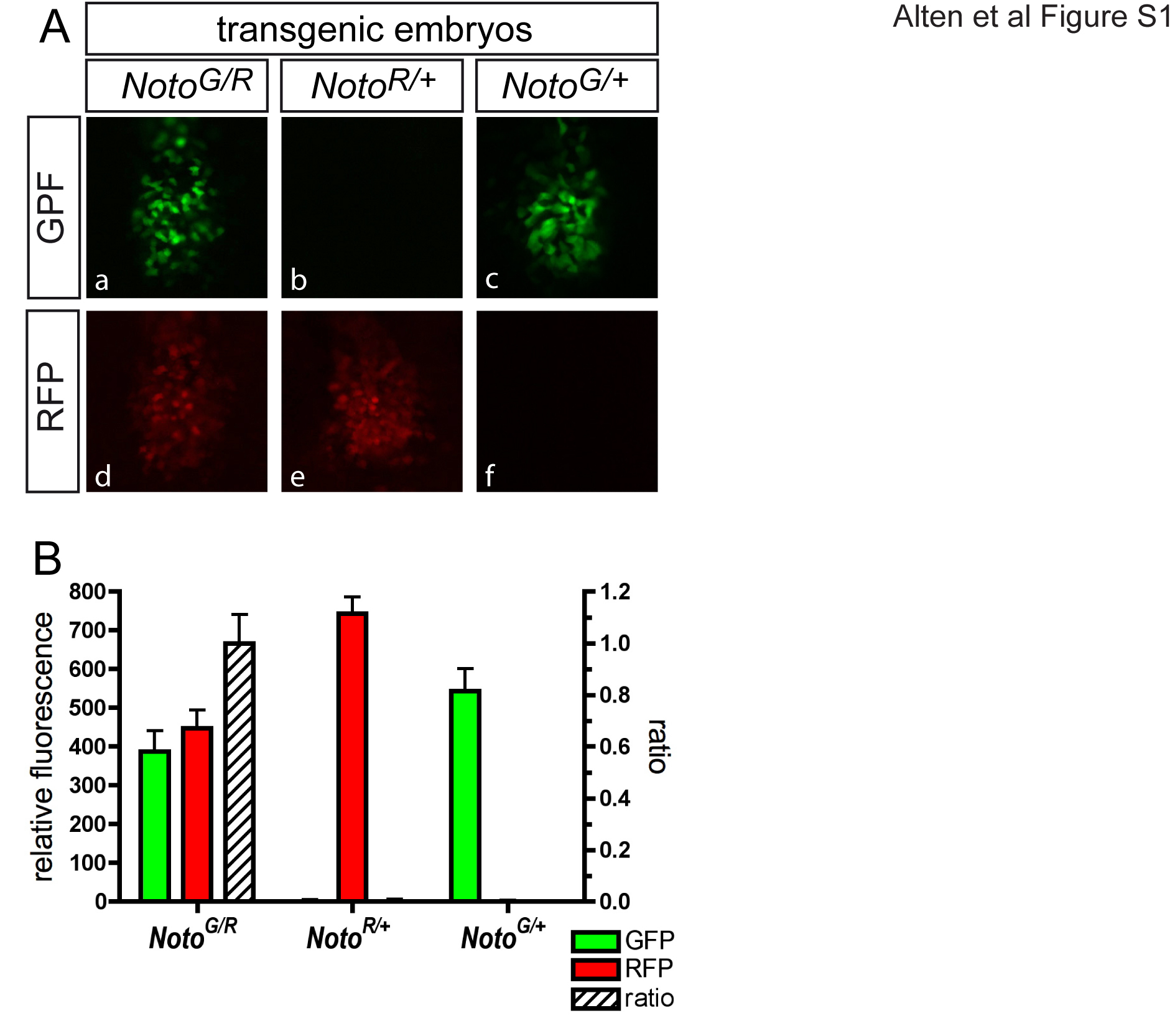

Supplement: Figure S1 — GFP and RFP fluorescence intensities in transgenic embryos. (A) GFP and RFP fluorescence in the node of NotoGFP/RFP (a, d), NotoRFP/+ (b, e) and NotoGFP/+ (c, f) transgenic embryos, (B) Fluorescence intensities of GFP and RFP and the ratio of GFP/RFP and RFP/GFP of transgenic embryos of the indicated genotypes (see also material and methods and Table S3). (JPG) [file pone.0047785.s001.jpg]

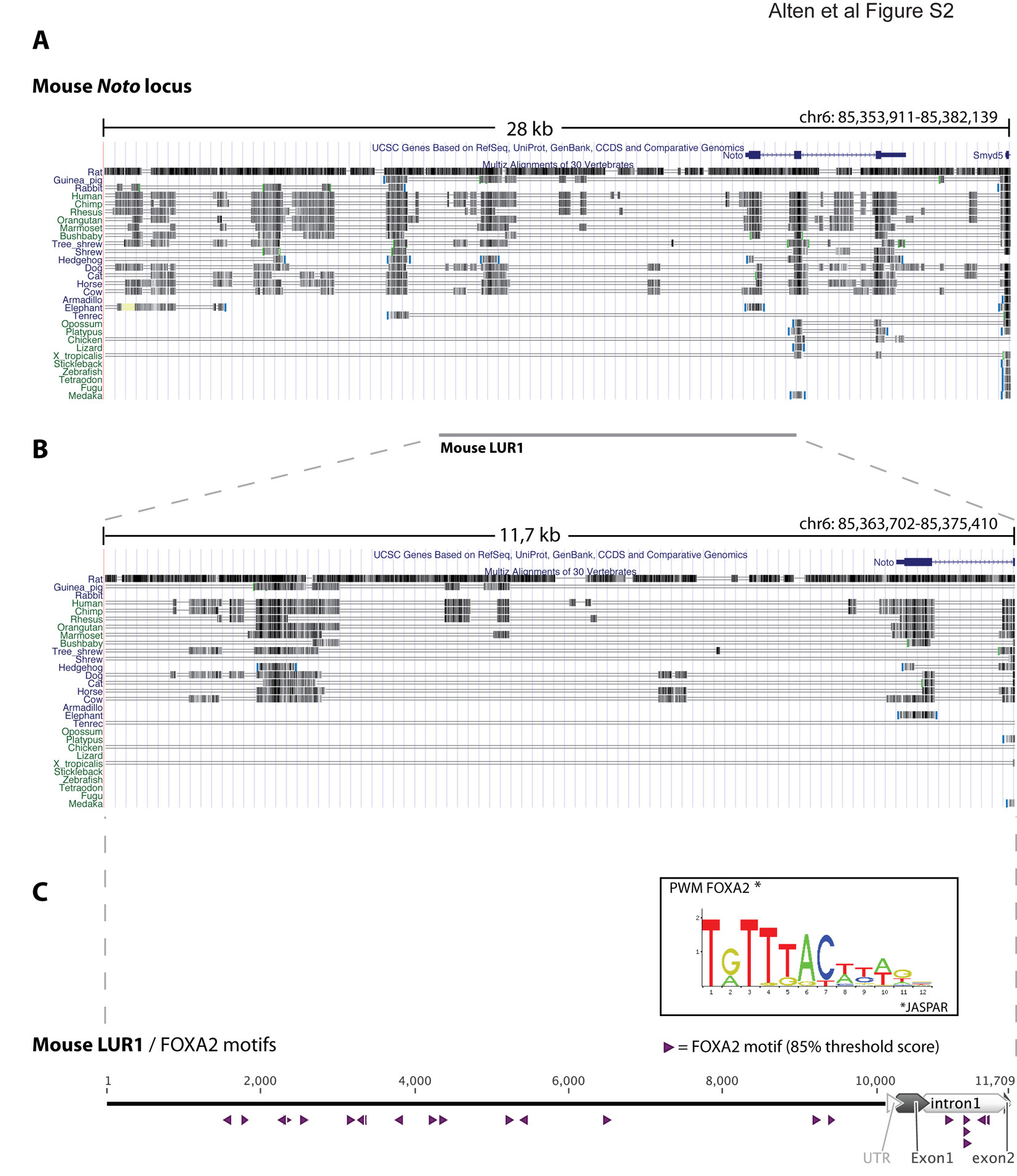

Supplement: Figure S2 — Conservation of the mouse Noto locus and construct LUR1. (A) Mouse Noto locus from the UCSC genome browser (Raney, Cline et al. 2011) with 30-way Multiz alignments (Blanchette, Kent et al. 2004) indicating conservation of sequences within vertebrate genomes. Genome coordinates from NCBI37/mm9 assembly (July 2007). (B) Genomic coordinates (mm9) of LUR1 reporter construct and 30-way Multiz alignments. (C) Position weight matrix logo for the transcription factor FOXA2 from the JASPAR database (Sandelin, Alkema et al. 2004) and location of putative FOXA2 binding sites in the LUR1 construct (85% threshold score). Annotation of UTR, exons and intron are adapted from Ensembl gene annotations. (JPG) [file pone.0047785.s002.jpg]

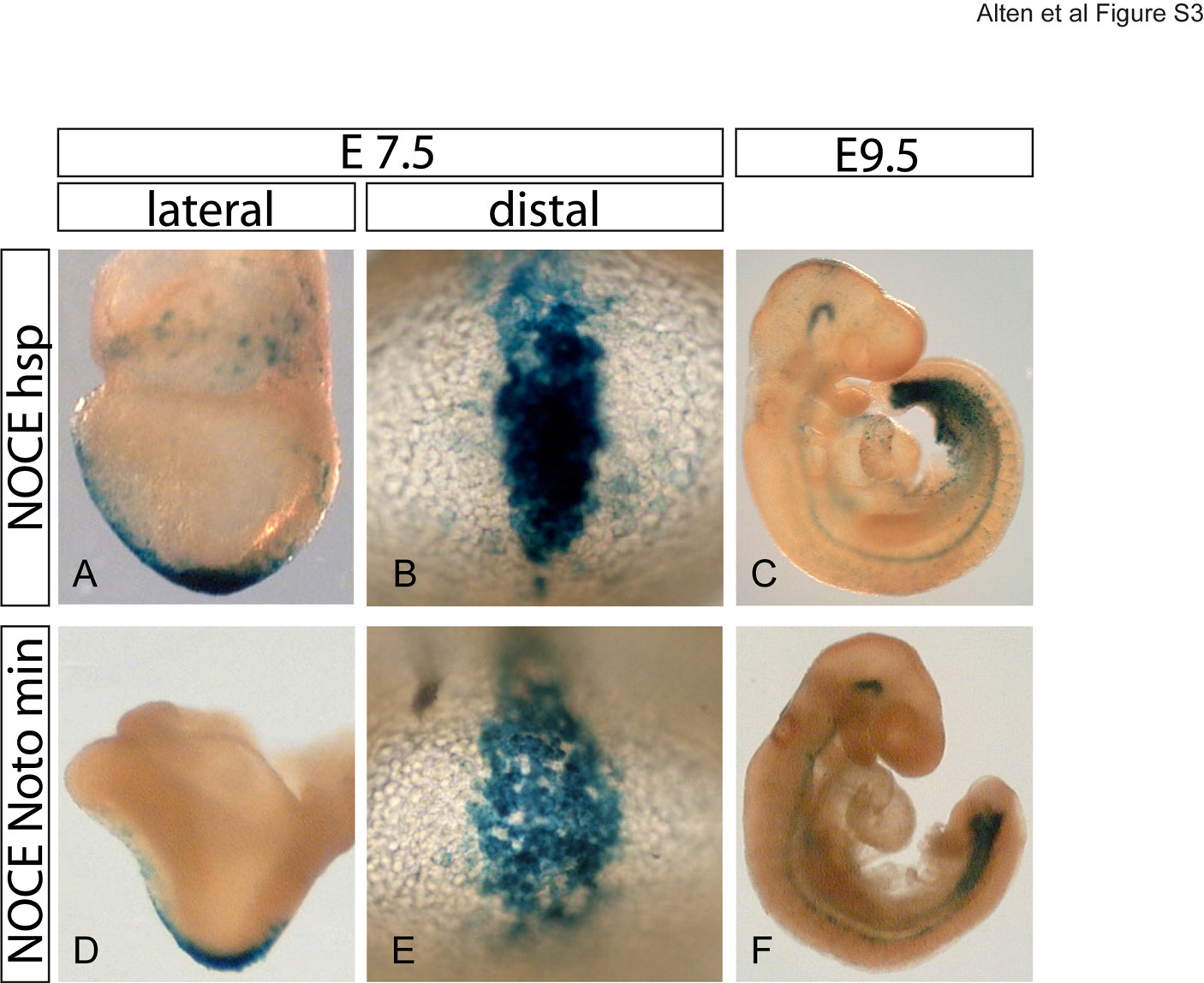

Supplement: Figure S3 — Comparison of reporter gene expression obtained by NOCE with the hsp and endogenous Noto minimal promoter. β-galactosidase staining of chimeric embryos carrying a promoter-reporter transgene of NOCE in front of the minimal promoter from hsp68 (A–C) or the endogenous Noto (D–F). (JPG) [file pone.0047785.s003.jpg]

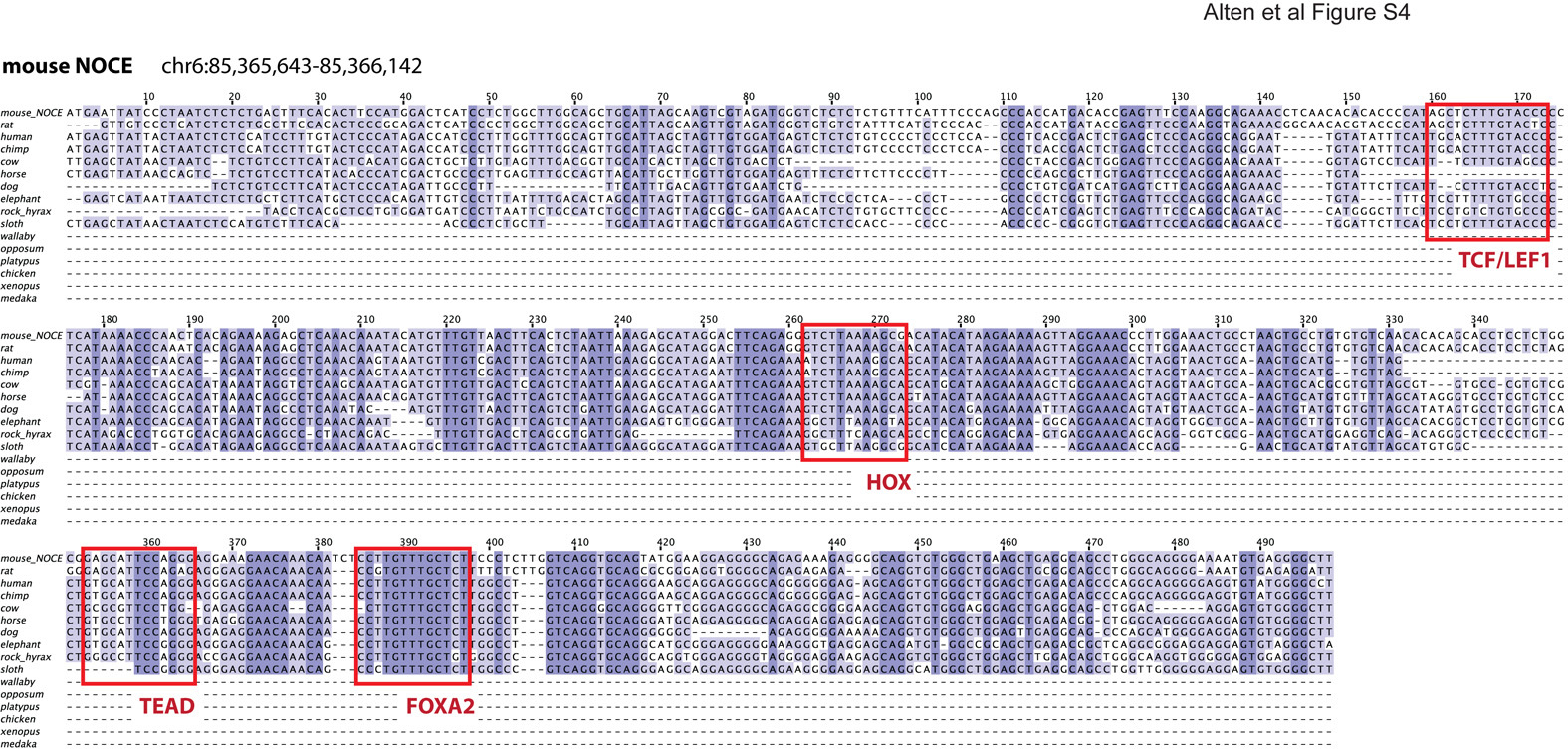

Supplement: Figure S4 — Alignment of mouse NOCE and orthologous sequences. Genomic coordinates of mouse NOCE (mm9), Multiz alignment of NOCE to orthologous sequences in representative species and annotation of putative binding sites for TCF/LEF1, TEAD, FOXA2 and HOX. No alignment can be found to species outside eutherian mammals, e.g. marsupial mammals, birds, amphibia and fish. The alignment was done by retrieving the 46-way Multiz hg19 alignments for selected species. (JPG) [file pone.0047785.s004.jpg]

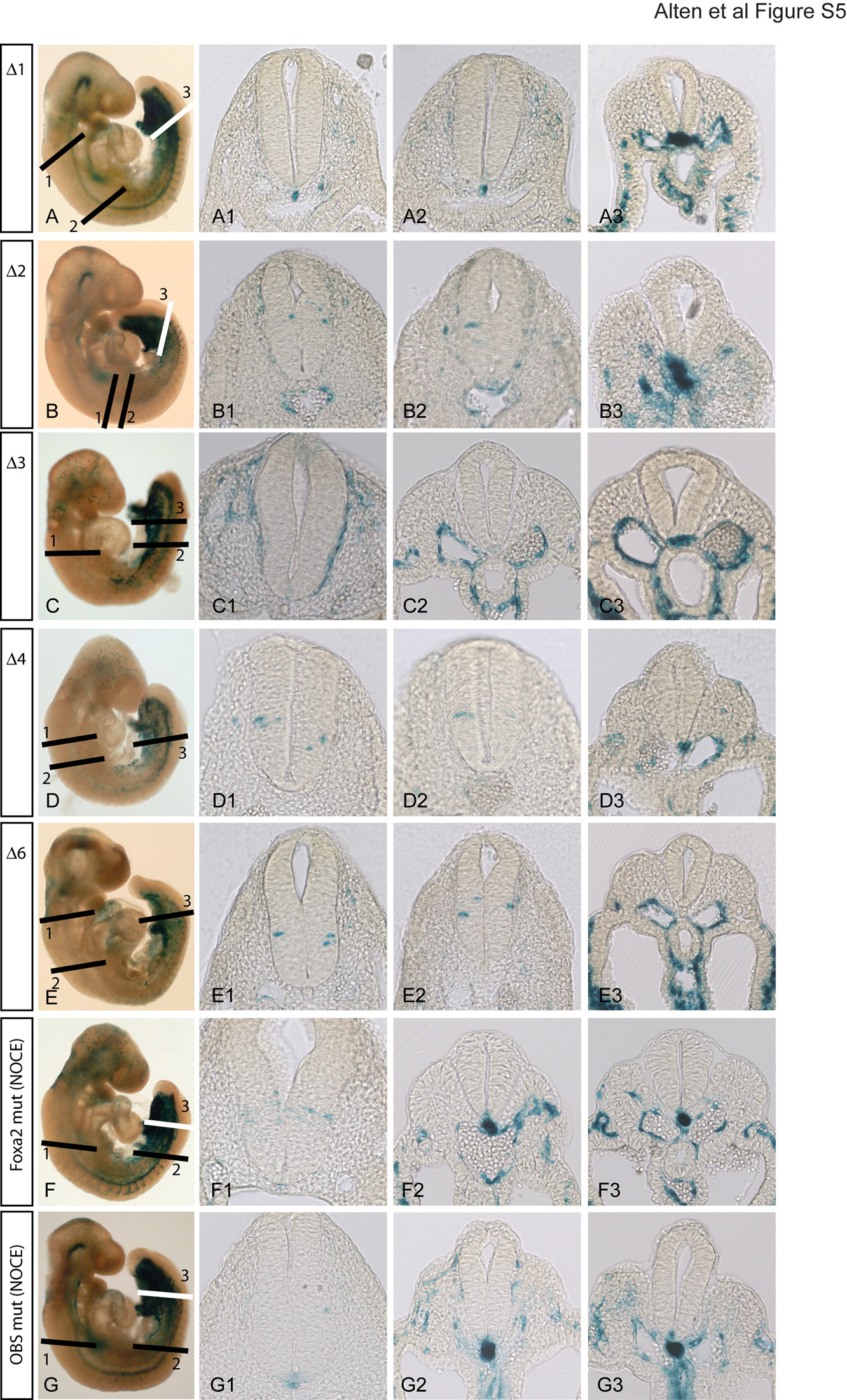

Supplement: Figure S5 — Gelatine sections of chimeric embryos after β-galactosidase staining. Genotypes are indicated on the left site. Lines indicate the respective section plane. (JPG) [file pone.0047785.s005.jpg]

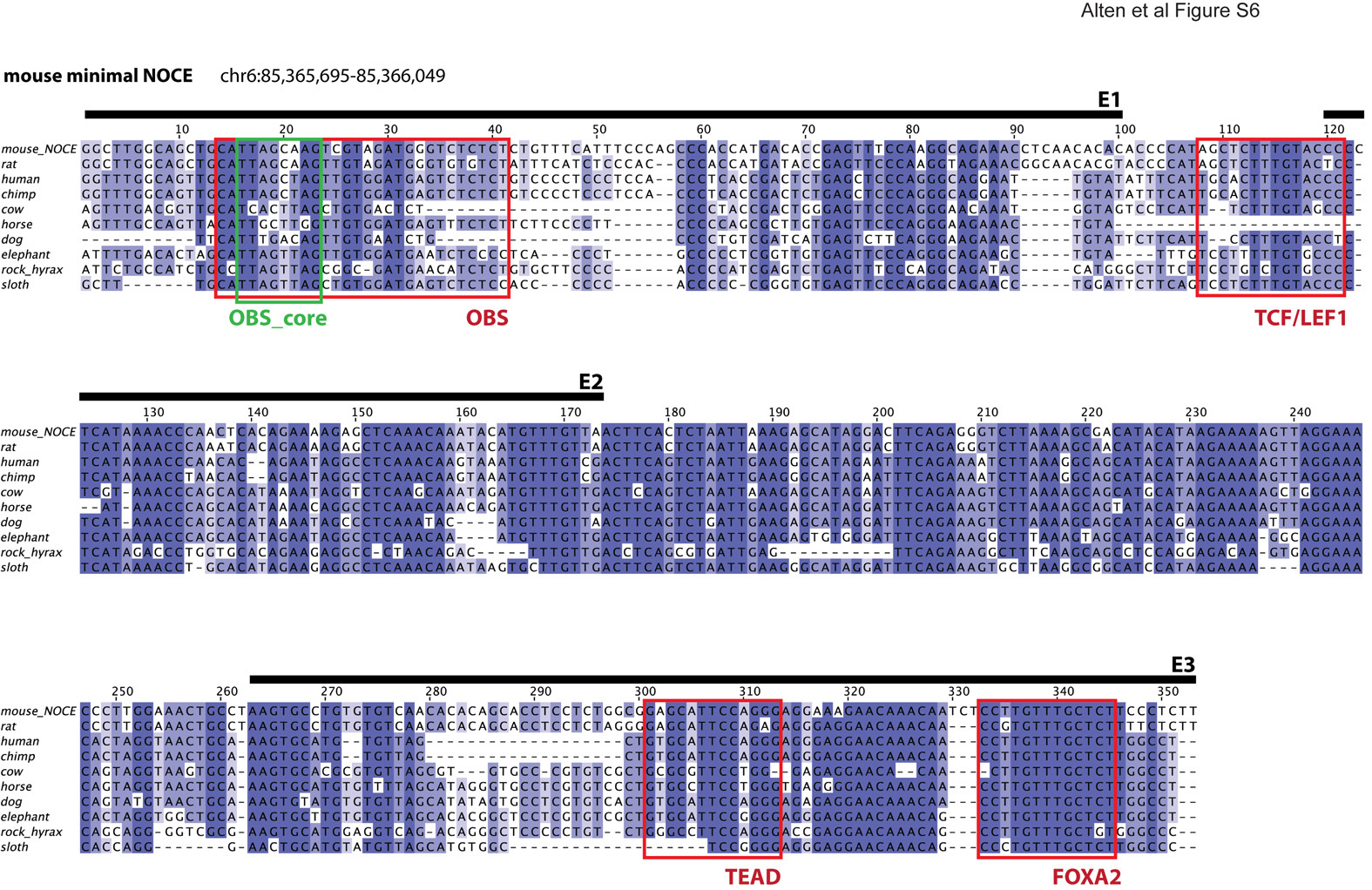

Supplement: Figure S6 — Annotated mouse NOCE core sequence. Genomic coordinates of mouse minimal-NOCE (mm9), Multiz alignment of the mouse sequence to orthologous sequences of representative eutherian mammalian species and annotation of putative binding sites OBS, OBS_core, TCF/LEF1, TEAD and FOXA2. The enhancer regions E1–E3 are indicated as black bars above the alignment. The alignment was done by retrieving the 46-way Multiz hg19 alignments for eutherian mammals. (JPG) [file pone.0047785.s006.jpg]

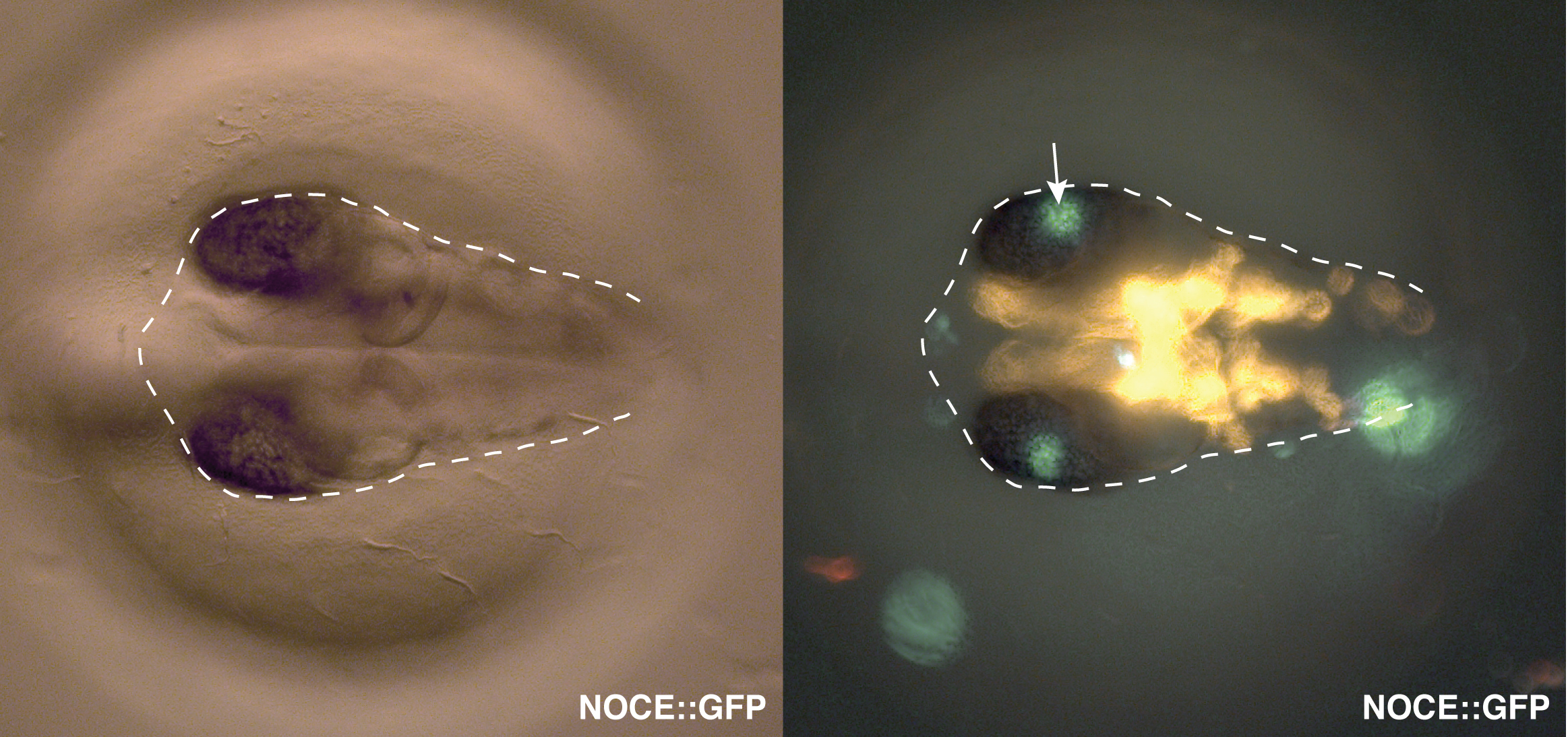

Supplement: Figure S7 — NOCE shows no specific enhancer activity in fish. Representative medaka embryo, transient transgenic with the NOCE::GFP reporter construct. The control expression in the lens (arrow) is attributed to the activity of the hsp70 promoter fragment that serves as technical control for successful genomic integration of the reporter. Additional green spots correspond to non-specific ectopic expression occurring in transient injected embryos. The observed autofluorescence (yellow patches) corresponds to the natural chromatophores in medaka fish. Live medaka stage 28 embryo is shown in dorsal view; anterior is oriented to the left. (JPG) [file pone.0047785.s007.jpg]
