# Supplementary material for: A Novel Mammal-Specific Three Partite Enhancer Element Regulates Node and Notochord-Specific Noto Expression
Source: PLoS One. 2012 Oct 22;7(10):e47785. doi: 10.1371/journal.pone.0047785 (PMC3478275; doi:10.1371/journal.pone.0047785)
Supplement: Table S1 — Sequences of primers used for ES cell screening and verification of promoter-reporter transgene insertions into the Hprt locus. (PDF) [file pone.0047785.s008.pdf]

Supplemental table 1: Sequences of primers used for ES cell screening and verification of promoter-reporter transgene insertions into the *Hprt* locus.

PCR for	Primer name	Sequence	Contructs
5‘ homology arm (after Hprt targeting)	Hprt 5‘typing-F1	TGAGTGGGGGGGTTGATAATCTTGG	all
	Noto-10-B1	AGTCCAGTTAGTGCTACACAGTGGG	LUR1, LUR2, LUR4, LUR5, TCF/Lef mutant, Hox mutant, Tead mutant
	Noto-7-B1	TTAAGTAGGGCACAGCCTGCAG	LUR3
	NOCE1-F	GCTAGCCTAGCCTGCTAGTA	NOCE rev
	del-B2	AAGCCCCTCACATTTTCCCC	NOCE, triple mutant, quadrupel mutant, Foxa2 and OBS single and double mutants, Δ3, 4
	NOCE-88-B	CCCATCTACGACTTGCTAATG	Δ1, 5, 6, 8, 9, 10
	NOCE-209-B	GCTCTTTTCTGTGAGTTGGG	Δ2, 7
3‘ homology arm (after Hprt targeting)	BAC3‘lacZ-F73	GGTAAACTGGCTCGGATTAGGG	all constructs
	pMP5 det-B	ATGTGTATGGGGGTTTCTGCTGC	
site-directed Mutagenesis	Foxa2-for	GAACAAACAATCTCCTGTCGACCTC TTCCCTCTTGGTC	Foxa2 binding site mutant constructs
	Tead-for	CACCTCCTCTGGCGGAGCCGGAAA GGGAGGAAAGAACAA	Tead binding site mutant constructs
	Hox-for	GAGCATAGGACTTCAGAGGGTCTGC AGAGCGACATACATAAGAAAAAG	Homeo domain binding site mutant constructs
	TCF/LEF-for	TCAACACACACCCCATAGCTTCTAG AACCCCCTCATAAAACCCAA	TCF/LEF binding site mutant constructs
	CEmut-for	TTGGCAGCTGCATGCTAGCCACGTA GATGGGT	OBS mutant constructs
5‘miniarm	5‘ miniarm-F1	TAACTAGTATCCAAAACACTGGCTG GTC	NOCE targeting construct
	5‘miniarm-B1	TAAAGCTTTCTAGTGGGGACATGGC TTC	
3‘miniarm	3‘miniarm-F1	TAAAGCTTAGGCAGGCAAAATCTCT GTG	
	3‘miniarm-B1	TAGGATCCGCTTTTATTTTGGTGGG GGT	
3‘ homology arm (after NOCE targeting)	pGKprom-B2	AGCTAGCTTGGCTGGACGTA	
	Noto-4-B3	CTGCCCAGAATTTCCTGAAG	
