# Supplementary material for: A Novel Mammal-Specific Three Partite Enhancer Element Regulates Node and Notochord-Specific Noto Expression
Source: PLoS One. 2012 Oct 22;7(10):e47785. doi: 10.1371/journal.pone.0047785 (PMC3478275; doi:10.1371/journal.pone.0047785)
Supplement: Table S2 — Number of analyzed chimeric embryos with various promoter-reporter transgenic ES cells. (PDF) [file pone.0047785.s009.pdf]

Supplemental table 2: Number of analyzed chimeric embryos with various promoter-reporter transgenic ES cells.

Transgene	embryos E7.5	embryos E9.5
LUR1	8	4
LUR2	8	3
LUR3	1	1
LUR4	3	4
LUR5	3	7
NOCE (hsp)	8	7
NOCErev	5	8
NOCE 3x mut	5	6
NOCE 4x mut	8	7
Δ1	6	4
Δ2	8	10
Δ3	4	5
Δ4	7	6
Δ5	3	4
Δ6	3	5
Δ7	2	1
Δ8	2	5
Δ9	2	2
NOCE Foxa2 mut	5	5
NOCE OBS mut	3	3
NOCE OBS+Foxa2 mut	3	5
NOCE Noto min	1	2

The developmental stage is indicated at the top and respective transgene is indicated at the left site.
