# Supplementary material for: A Novel Mammal-Specific Three Partite Enhancer Element Regulates Node and Notochord-Specific Noto Expression
Source: PLoS One. 2012 Oct 22;7(10):e47785. doi: 10.1371/journal.pone.0047785 (PMC3478275; doi:10.1371/journal.pone.0047785)
Supplement: Table S3 — Fluorescence intensities and ratios of transgenic and chimeric embryos. (PDF) [file pone.0047785.s010.pdf]

Supplemental table 3: Fluorescence intensities and ratios of transgenic and chimeric embryos.

	transgenic embryos			chimeric embryos		
	$Noto^{G/R}$	$Noto^{R/+}$	$Noto^{G/+}$	$Noto^{G/R}$	$Noto^{G\Delta N/R}$	$Noto^{G/R\Delta N}$
number of embryos	10	13	5	13	30	38
average GFP fluorescence	496,50	109,80	653,00	502,60	139,30	871,10
corrected average GFP fluorescence	386,70	0	543,20	392,84	29,49	761,30
average RFP fluorescence	505,80	801,20	58,40	548,80	499,40	64,26
corrected average RFP fluorescence	447,40	742,80	0	490,44	441,03	5,86
ratio of fluorescences	GFP/RFP	GFP/RFP	RFP/GFP	GFP/RFP	GFP/RFP	RFP/GFP
	0,8643	0	0	0,800995	0,066866	0,007697
correction factor	/0,8643	/0,8643	*0,8643	/0,8643	/0,8643	*0,8643
normalized ratio	1	0	0	0,9266	0,0773	0,0066

The table indicates the numbers of analyzed transgenic and chimeric embryos of each genotype, averages of the measured fluorescence intensities of each group, background-corrected average values, the ratios and normalized ratios of the intensities of the GFP and RFP alleles as indicated.
